# Supplementary material for: Effects of voluntary event cancellation and school closure as countermeasures against COVID-19 outbreak in Japan
Source: PLoS One. 2020 Dec 21;15(12):e0239455. doi: 10.1371/journal.pone.0239455 (PMC7751859; doi:10.1371/journal.pone.0239455)
Supplement: S1 File — (DOCX) [file pone.0239455.s001.docx]

Data sources in 46 prefectures and some cities

https://www.pref.okinawa.lg.jp/site/hoken/chiikihoken/kekkaku/covid19_hasseijoukyou.html

https://www.pref.kagoshima.jp/ae06/kenko-fukushi/kenko-iryo/kansen/kansensho/coronavirus.html

https://www.pref.miyazaki.lg.jp/kansensho-taisaku/covid-19/hassei_list.html https://www.pref.oita.jp/site/covid19-oita/covid19-pcr.html https://www.pref.kumamoto.jp/kiji_32300.html https://www.pref.nagasaki.jp/bunrui/hukushi-hoken/kansensho/corona_nagasaki/corona_nagasaki_shousai/

https://www.pref.saga.lg.jp/kiji00373220/index.html https://www.pref.fukuoka.lg.jp/contents/covid19-hassei.html https://www.city.fukuoka.lg.jp/hofuku/hokenyobo/health/kansen/cohs.html https://www.pref.kochi.lg.jp/soshiki/130401/2020022900049.html https://www.pref.ehime.jp/h25500/kansen/covid19.html#kansensha https://www.pref.kagawa.lg.jp/content/dir1/dir1_6/dir1_6_2/wt5q49200131182439.shtml#outbreak https://www.pref.yamaguchi.lg.jp/cms/a10000/korona2020/202004240002.html https://www.pref.hiroshima.lg.jp/soshiki/57/bukan-coronavirus.html https://www.pref.okayama.jp/page/645925.html#kennaijoukyou https://www.pref.shimane.lg.jp/bousai_info/bousai/kikikanri/shingata_taisaku/new_coronavirus_portal.html

https://www.pref.tottori.lg.jp/291425.htm https://www.pref.wakayama.lg.jp/prefg/041200/d00203387.html http://www.pref.nara.jp/module/1356.htm#moduleid1356 https://web.pref.hyogo.lg.jp/kk03/corona_hasseijyokyo.html http://www.pref.osaka.lg.jp/hodo/index.php?HST_TITLE1=%83R%83%8D%83i&SEARCH_NUM=10&searchFlg=%8C%9F%81@%8D%F5&site=fumin https://www.pref.kyoto.jp/kentai/news/novelcoronavirus.html#F https://www.pref.shiga.lg.jp/ippan/kenkouiryouhukushi/yakuzi/310735.html https://www.pref.mie.lg.jp/YAKUMUS/HP/m0068000066.htm https://www.pref.aichi.jp/site/covid19-aichi/corona-kisya.html http://www.city.nagoya.jp/kenkofukushi/page/0000126920.html https://www.pref.shizuoka.jp/kinkyu/covid-19-tyuumokujouhou.html https://www.pref.gifu.lg.jp/kinkyu-juyo-joho/shingata_corona_kansendoko.html https://www.pref.nagano.lg.jp/hoken-shippei/kenko/kenko/kansensho/joho/corona-doko.html https://www.pref.yamanashi.jp/koucho/coronavirus/info_coronavirus_prevention.html https://www.pref.fukui.lg.jp/doc/kenkou/corona/jyoukyou.html https://www.pref.ishikawa.lg.jp/kansen/coronakennai.html http://www.pref.toyama.jp/cms_sec/1205/kj00021798.html https://www.pref.niigata.lg.jp/sec/kenko/covid19.html https://www.pref.kanagawa.jp/docs/ga4/bukanshi/occurrence_06.html https://www.bousai.metro.tokyo.lg.jp/taisaku/saigai/1007261/index.html https://www.pref.chiba.lg.jp/shippei/press/2019/ncov-index.html https://www.pref.saitama.lg.jp/a0701/shingatacoronavirus.html https://www.pref.gunma.jp/07/z87g_00016.html http://www.pref.tochigi.lg.jp/e04/welfare/hoken-eisei/kansen/hp/coronakensahasseijyoukyou.html

https://www.pref.ibaraki.jp/1saigai/2019-ncov/hassei.html https://www.pref.fukushima.lg.jp/sec/21045c/fukushima-hasseijyoukyou.html https://www.pref.yamagata.jp/ou/bosai/020072/kochibou/coronavirus/coronavirus.html#kensa

https://www.city.yamagata-yamagata.lg.jp/kakuka/kenkoiryo/kenkozoshin/sogo/kansensyou/pd0409180023.html

https://www.pref.akita.lg.jp/pages/archive/47957 https://www.pref.miyagi.jp/site/covid-19/02.html https://www.pref.iwate.jp/kurashikankyou/iryou/covid19/index.html https://www.pref.aomori.lg.jp/welfare/health/wuhan-novel-coronavirus2020.html http://www.pref.hokkaido.lg.jp/hf/kth/kak/hasseijoukyou.htm#4/12
